# HeartMate 3 implantation for dextro‐transposition of the great arteries after Mustard procedure: A technique of papillary muscle repositioning

**DOI:** 10.1111/jocs.16970

**Published:** 2022-10-02

**Authors:** Albert C. Pai, Anthony L. Panos, Marco Ricci

**Affiliations:** ^1^ Department of Cardiothoracic Surgery University of Iowa Hospitals and Clinics Iowa City Iowa USA; ^2^ Division of Pediatric Cardiac Surgery, Stead Family Children's Hospital Iowa City Iowa USA

**Keywords:** HeartMate 3, Mustard procedure, papillary muscle repositioning

## Abstract

Systemic right ventricular failure after physiologic repair for dextro‐transposition of the great arteries can be managed with durable mechanical circulatory support; however, the right ventricular morphology, such as intervening papillary muscles, presents challenges to inflow cannula positioning. Papillary muscle repositioning is an innovative technique to circumvent obstructive anatomy.

## 1 INTRODUCTION

**Figure 1:**
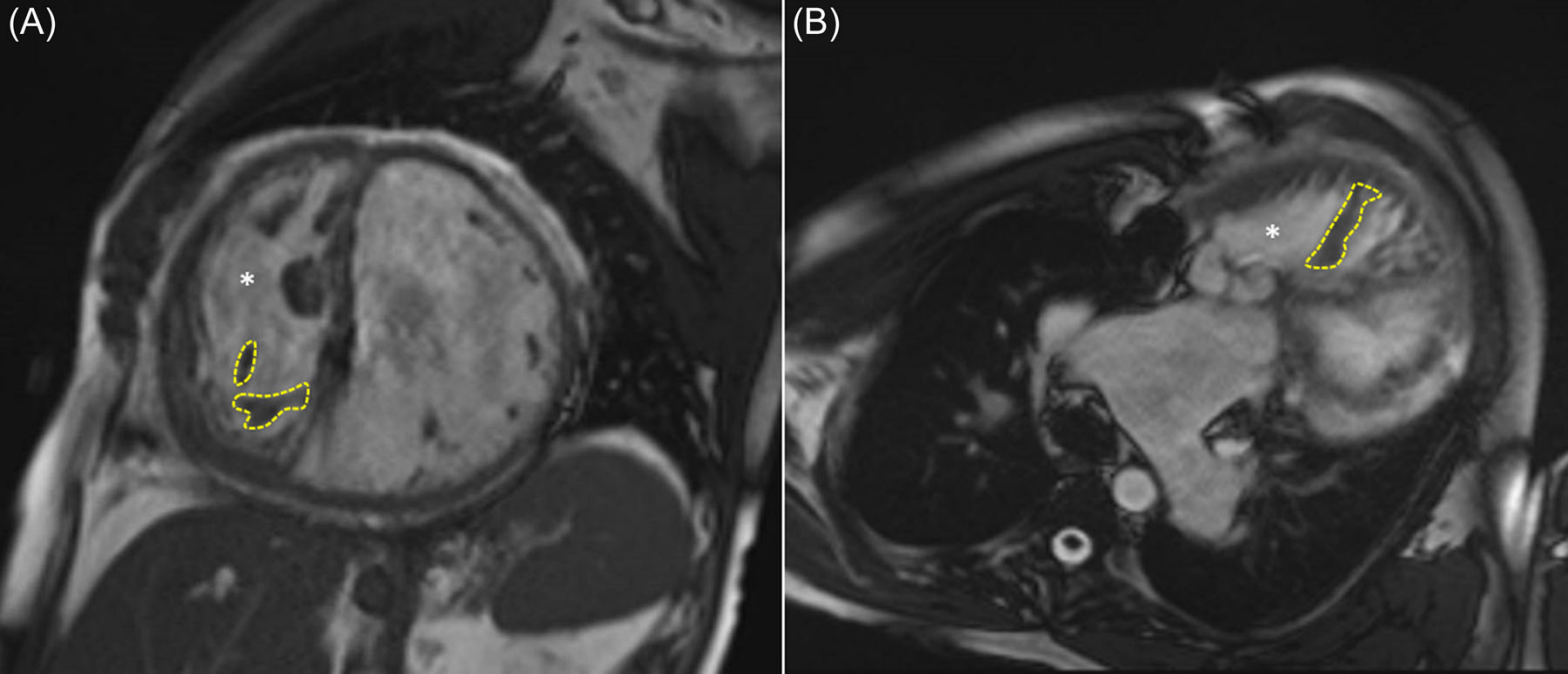
Cardiac MRI characterizing right ventricular morphology. (A) Short‐axis view seen from a sagittal cut shows a dilated RV and LV. *indicates the RV. The structure outlined in dotted yellow are the papillary muscles along the diaphragmatic free wall of the RV. (B) A representative slice of the four‐chamber view from a transverse cut. MRI, magnetic resonance imaging; LV, left ventricle; RV, right ventricle.

A 43‐year‐old male with a history of dextro‐transposition of the great arteries (D‐TGA) status post‐Mustard palliation at 6 months of age presented with acute exacerbations of systolic heart failure (New York Heart Association class IV) over 3 months with the latest admission involving a non‐ST elevation myocardial infarction. Cardiac catheterization demonstrated low cardiac output (1.4 L/min) and elevated pulmonary capillary wedge pressure (24 mmHg). While admitted, his clinical condition deteriorated to Interagency Registry for Mechanically Assisted Circulatory Support level 2 with progressive decline of hemodynamics despite inotropic support. Cardiac magnetic resonance imaging revealed severe global systolic function and calculated an end‐diastolic volume index of 164 ml/m^2^ and a right ventricular ejection fraction of 29% (Figure 1). He was indicated for a surgically implanted mechanical circulatory support (MCS) in the systemic right ventricle (RV), specifically a HeartMate III (HM3), for destination therapy. The anatomy of a hypertrophic and dilated RV presents challenges to inflow cannula implantation. Here, we describe a technique of papillary muscle repositioning to facilitate RV assist device insertion.

## 2 METHOD

### 2.1 Detailed surgical technique

**Figure 2:**
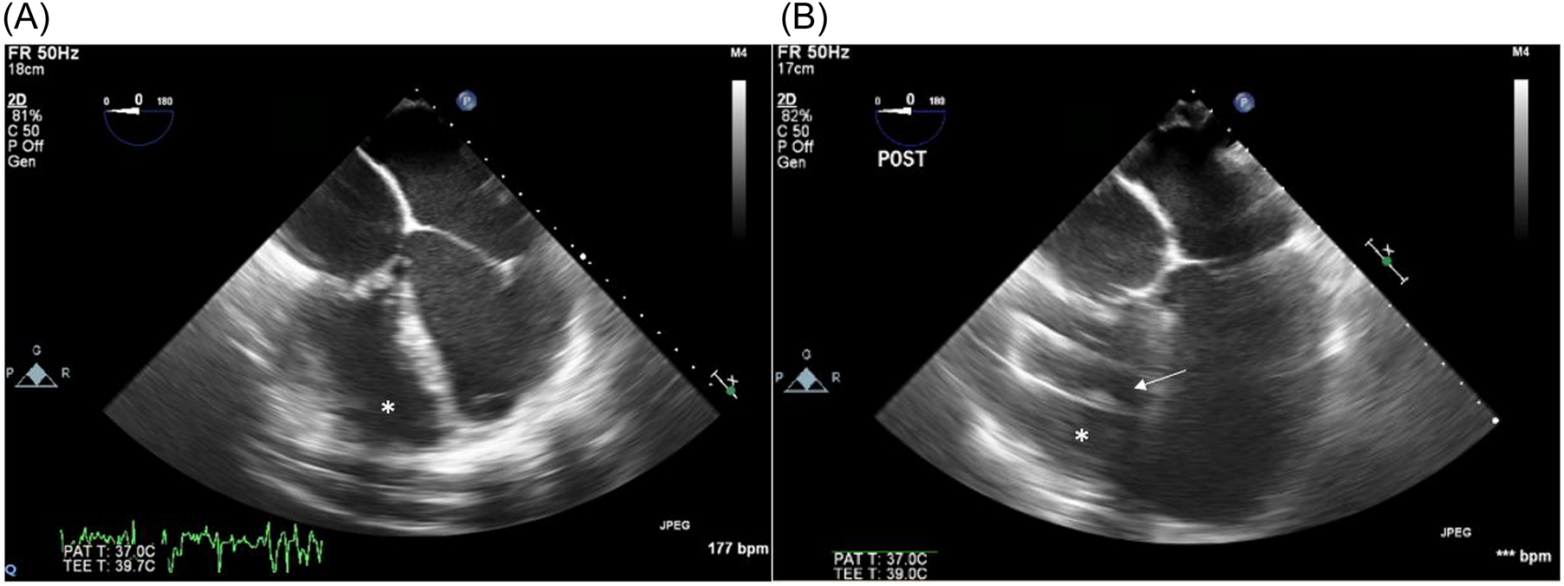
TEE images. (A) Preoperative four‐chamber view. *indicates the RV. (B) Postoperative four‐chamber view. The white arrow indicates the inflow cannula directed toward the right ventricular outflow tract. RV, right ventricle; TEE, transesophageal echocardiogram.

**Figure 3:**
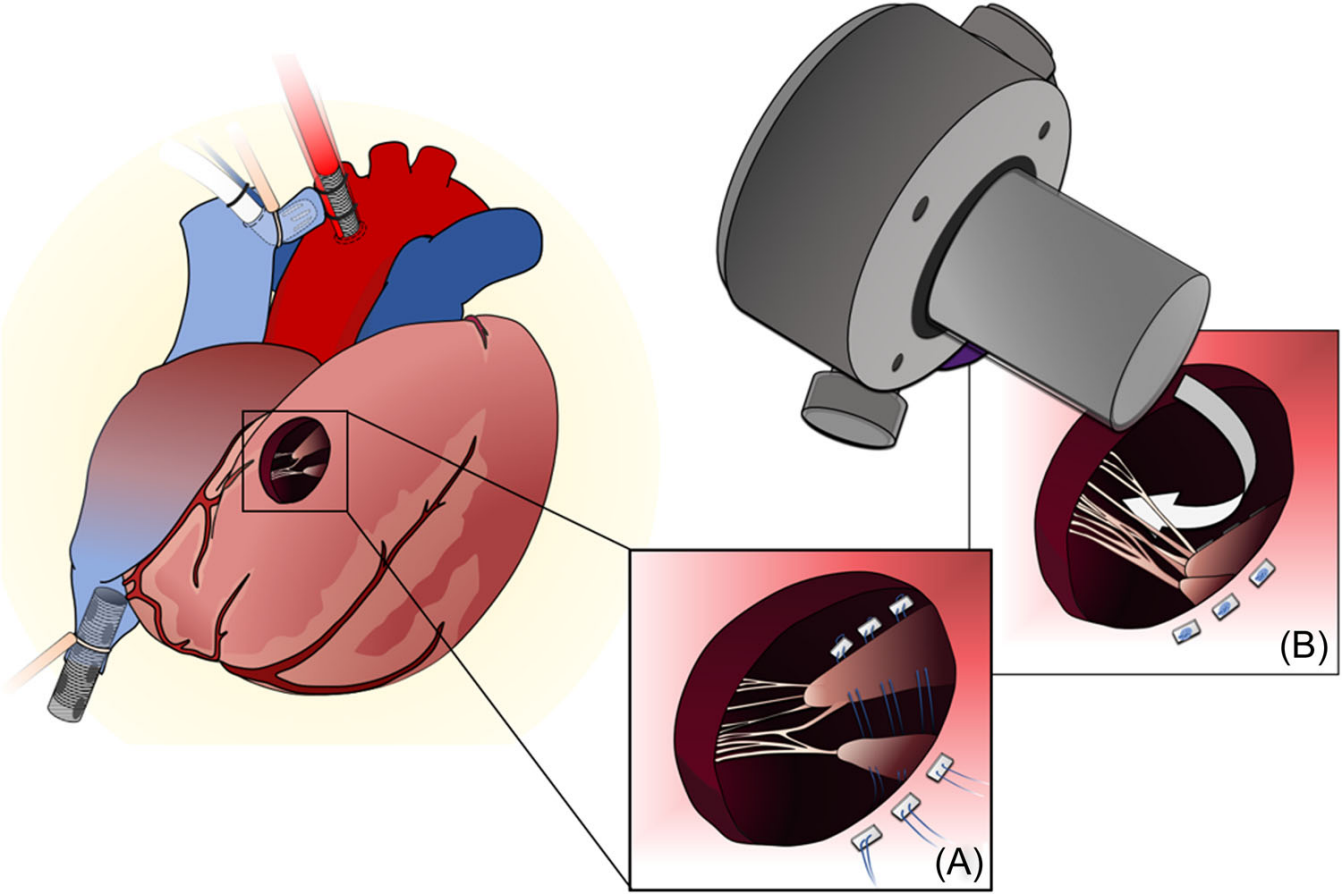
Papillary muscle repositioning. (A) Pledgetted 4‐0 Prolene suture anchors the papillary muscle to the right ventricular free wall. (B) The path between the atrioventricular valve to the inflow cannula is now unobstructed.

After redo sternotomy, the aorta was cannulated centrally, and bicaval venous cannulation was achieved via the femoral vein and left innominate vein. The anatomy was confirmed to be that of D‐TGA with an anterior, rightward aorta originating from the RV. We selected a site on the diaphragmatic surface of the RV near the acute marginal border for inflow insertion, and transesophageal echocardiography (TEE) guidance revealed a heavily trabeculated RV (Figure 2A). To ensure appropriate positioning of the inflow cannula within the ventricle, we opted to arrest the heart. We then cored the ventricle to confirm two broad papillary muscles with attached chordae tendineae obscuring our view of the RV long axis. We placed a series of horizontal mattress stitches using pledgeted 4‐0 Prolene suture through the papillary muscles and out through the inferior wall of the RV. This anchored the papillary muscle out of the cannula's path (Figure 3). Additional intervening trabeculae without chordal attachments were sharply excised. The apical connector cap was secured to the RV wall with full‐thickness horizontal mattress sutures using 2‐0 Ethibond. The cross‐clamp was released after adequate deairing. In our usual fashion, the ventricular assist device (VAD) outflow graft was then draped around the right atrium and anastomosed to the ascending aorta with a partial side‐biting clamp. TEE at the conclusion of the case showed the inflow cannula directed toward the right ventricular outflow tract (Figure 2B) and confirmed the absence of tricuspid stenosis (TS) with notable moderate tricuspid regurgitation. The postoperative hemodynamic parameters were as follows: 5400 rpm, flow 4.5 L/min, pulsatility index 3.1, power 3.5 W. Anticoagulation via heparin infusion was initiated on postoperative Day 3 to a therapeutic level.

## 3 COMMENT

We describe the unique presentation of HM3 implantation within a systemic RV for D‐TGA after Mustard palliation. While the atrial switch is now uncommonly performed as the principal operation, there are long‐term survivors who inevitably develop systemic RV failure mostly due to an inability of the morphologic RV to sustain systemic pressures.^1^, ^2^, ^3^ As in LV failure, durable MCS should be a treatment strategy for refractory systemic RV failure.

HM3 implantation in the LV apex requires the inflow cannula to be inserted parallel to the interventricular septum and directed toward the atrioventricular valve. This can typically be performed without cardioplegic arrest and with reliance on TEE guidance. In our D‐TGA scenario, there are several anatomic considerations that make apical implantation technically challenging. The morphologic RV is more tubular as opposed to the cone‐shaped LV, and the less‐developed RV apex, especially one that is hypertrophic or dilated, may not necessarily correspond to the apex of the heart. As such, insertion points can vary from the anterior to the diaphragmatic RV. The RV is also relatively more trabeculated, and the abundance of muscle bundles and papillary muscles can interfere with device placement and subsequent inflow drainage.

For these reasons, we advocate for cardioplegic arrest to decompress the heart and position the device under direct vision. Excision of trabeculations and the moderator band have been reported,^4^, ^5^, ^6^ but the management of intersecting papillary muscle has not been described in this setting. Papillary muscle repositioning is a technique that is utilized for mitral^7^ or tricuspid^8^ valve repair. Suture redirection of the papillary head changes the vector of chordal attachments and of the atrioventricular valve leaflets while preserving the ventricular geometry. We use this method because the versatility of the technique allows the surgeon to reposition the papillary muscles in any direction to accommodate unpredictable VAD positioning. The one caveat is the possibility of functional TS, which can affect device inflow. It is critical to confirm minimal TS at the conclusion of the procedure. Tricuspid regurgitation will have limited clinical impact due to inflow suction limiting regurgitant volume.

**Figure 4:**
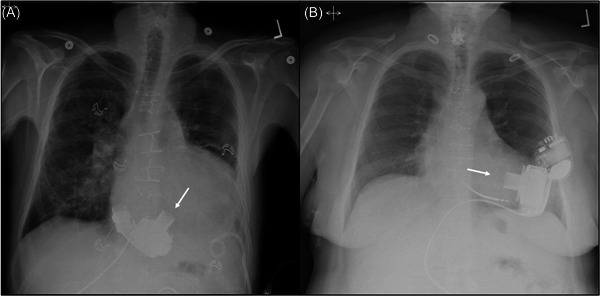
HeartMate 3 positioning comparison. (A) Right ventricular placement with the arrow indicating the positioning of the inflow cannula. (B) Typical left ventricular placement of the LV assist device).

The patient recovered well and was discharged on postoperative Day 43. Positioning of the device on chest roentgenogram can be compared to that of “normal” left ventricular (LV) positioning (Figure 4).

## CONFLICT OF INTEREST

The authors declare no conflict of interest.

## ETHICS STATMENET

We waive the option to obtain consent as the following writing involves no more than minimal risk to the patient. This waiver will not affect the rights and welfare of the described subject. If appropriate, the patient will be provided with additional pertinent information gained from the readership.
